# rTMS in the Treatment of Drug Addiction: An Update about Human Studies

**DOI:** 10.1155/2014/815215

**Published:** 2014-01-23

**Authors:** Elisa Bellamoli, Paolo Manganotti, Robert P. Schwartz, Claudia Rimondo, Maurizio Gomma, Giovanni Serpelloni

**Affiliations:** ^1^Addiction Department, ULSS 20, Via Germania 20, 37136 Verona, Italy; ^2^Department of Neurological and Movement Sciences, University of Verona, Piazzale L.A. Scuro 10, 37134 Verona, Italy; ^3^Friends Research Institute, Inc., 1040 Park Avenue, Suite 103, Baltimore, MD 21201, USA; ^4^National Coordination Centre for NIDA Collaborations, Via Germania 20, 37136 Verona, Italy; ^5^' Department for Anti-Drug Policies of the Presidency of Ministers' Council of Italy, Via dei Laterani 34, 00184 Roma, Italy

## Abstract

Drug addiction can be a devastating and chronic relapsing disorder with social, psychological, and physical consequences, and more effective treatment options are needed. Repetitive transcranial magnetic stimulation (rTMS) is a noninvasive brain stimulation technique that has been assessed in a growing number of studies for its therapeutic potential in treating addiction. This review paper offers an overview on the current state of clinical research in treating drug addiction with rTMS. Because of the limited research in this area, all studies (including case reports) that evaluated the therapeutic use of rTMS in nicotine, alcohol, or illicit drug addiction were included in this review. Papers published prior to December 2012 were found through an NCBI PubMed search. A total of eleven studies were identified that met review criteria. There is nascent evidence that rTMS could be effective in reducing cocaine craving and nicotine and alcohol craving and consumption and might represent a potential therapeutic tool for treating addiction. Further studies are needed to identify the optimal parameters of stimulation for the most effective treatment of drug addiction, to improve our comprehension of the treatment neurophysiological effects, and to conduct rigorous, controlled efficacy studies with adequate power.

## 1. Introduction

Psychoactive drugs act on the central nervous system and recurring drug intoxication can result in addiction, a complex disease process of the brain which can be treated [1]. Addiction can be described as a persistent state in which there is reduced capacity to control compulsive drug-seeking, regardless of whether it involves risk of negative consequences [2, 3]. It is often a devastating and chronically relapsing disorder with social, psychological, and physical consequences. Drug addiction incurs enormous medical, economic, and social costs. The currently available treatment options for addiction remain somewhat limited and long-term success rates are modest [4].

Brain stimulation allows modulation of activity in specific brain regions. Recently, nonsurgical brain stimulation techniques have been utilized in examining the effects of drug administration on cortical activity in order to further explore the effects of repeated drug use on cortical excitability. Furthermore, several novel studies have begun to assess brain stimulation as a potential treatment for reducing addictive behaviours [5].

This review paper offers an overview on the current state of research in treating addiction in humans with transcranial magnetic stimulation (TMS), a noninvasive brain stimulation technique that may have therapeutic potential in treating addiction.

Due to the limited amount of research in this area, all studies (including case reports) that were identified through an NCBI PubMed search (http://www.ncbi.nlm.nih.gov/) were included in the review if published prior to December 2012 and if reporting on the evaluation of the therapeutic use of rTMS in tobacco, alcohol, or illicit drug addiction. Search terms included “repetitive transcranial magnetic stimulation,” and “rTMS,” with adjoining terms “addiction,” “drug,” “nicotine,” “tobacco,” “alcohol,” “cocaine,” “opioids,” “heroin,” “cannabis,” “marijuana,” “MDMA,” and “ecstasy.” A total of 11 studies were identified and included in this review and were classified according to the system reported by Brainin et al. [6] for the European Federation of Neurological Societies. In this system, individual studies are rated on a continuum based on the level of evidence as follows: class I requires a rigorously conducted randomized clinical trial (RCT); class II requires a less rigorously conducted RCT or a prospective matched-group cohort study; class III requires any other type of controlled trial; and class IV requires uncontrolled studies, case series, case reports, or expert opinion. Based on the evidence, the approaches are then rated in three levels: level A indicates the approach is effective, ineffective, or harmful if there is at least one class I study or two class II studies; level B indicates the approach is probably effective, ineffective, or harmful if there is at least one convincing class II study or overwhelming class III evidence; and level C indicates the approach is possibly effective, ineffective, or harmful if there are at least two convincing class III studies.

## 2. Transcranial Magnetic Stimulation 

TMS is a nonsurgical brain stimulation technique that is proving to be valuable for both its research and therapeutic potential within psychiatry [7]. TMS is able to modulate cortical excitability and it is used to facilitate functional brain mapping of cortical regions [8]. It uses a magnetic pulse of high intensity, focused in a limited area, which is administered through a coil. The extremely fast passage of electric current in the coil induces a transient, high-intensity magnetic pulse that penetrates through the scalp and reaches the underlying cortex. In the targeted cortical area, the magnetic pulse generates an electric current that induces depolarization of superficial cortical neurons [9]. In the region beneath the coil and interconnected areas a stimulation occurs or a disruption of local neural activity [10, 11].

Repetitive TMS (rTMS) is used to induce longer lasting alterations, facilitation, or functional disruptions. In rTMS, trains of several pulses are delivered using various stimulation patterns [12]. It provides a repeated stimulation of the scalp at the same point with a frequency ranging from 1 to 20 Hz or more. The parameters in rTMS include its intensity, frequency, length of trains of pulses, and time interval between trains. The effects of rTMS are longer than TMS single pulse. These long-term changes in the functioning of the cortex produce effects that vary depending on the frequency of stimulation, resulting in inhibition or facilitation.

While the long-lasting neurophysiologic effects of rTMS are poorly understood, several studies showed that rTMS can induce significant and long-lasting behavioural alterations, including reduction in craving and consumption of drugs of abuse.

The safety of TMS has been reported in a number of studies [13–15] and the most recent guidelines for its use have been published in 2009 by Rossi and colleagues [16]. The use of TMS is generally not recommended with patients who have a history of epilepsy, in carrying a pacemaker, with hearing aids, and with metal in any part of the body and in pregnant women. Single pulses or low frequency stimulation appears to carry little risk beyond occasionally causing local discomfort at the site of stimulation. In some circumstances, especially when the intensity of the magnetic pulse is high, the effect of current induced by the magnetic field can spread to the surrounding muscles on the head and neck, causing contractions. Occasionally, mild or moderate transient headache has been reported. Long-term effects of repeated rTMS sessions are as yet unknown. Based on existing data, rTMS appears safe when administered according to recommended guidelines, and its safety record supports its further development as a clinical treatment [15, 17].

## 3. TMS and Cortical Excitability in Addiction: Physiopathological Mechanisms

There were some reports of using TMS to measure cortical inhibition in the cerebral cortex of subjects with substance dependence [18–28]. These studies found that chronic exposure to various addictive drugs induces alterations in cortical excitability in the motor, occipital, and prefrontal cortex (PFC). Cortical conductivity has been evaluated in individuals with exposure to alcohol [18–22], with nicotine dependence [23], with chronic cannabis exposure [24], with chronic ecstasy exposure [25], and with cocaine addiction [26–28].

Decreased excitability in the motor cortex was found in individuals who are cocaine [26–28] and nicotine dependent [23]. Chronic cannabis use is associated with a reduction in cortical inhibition [24]. Increased excitability of the visual cortex was found in individuals with chronic MDMA use, abstinent for 3 days [25]. Acute alcohol exposure was found to be associated with decreased excitability in the motor cortex [18, 19] and the PFC [20]. Moreover, Conte et al. [21] proposed that while acute ethanol administration seemed to affect GABA neurotransmission, chronic administration appeared to alter the glutamate mechanisms involved in cortical excitability. Nardone and colleagues (2010) found that TMS showed a selective increase in intracortical facilitation after ethanol withdrawal which supported the theory that altered glutamatergic receptor function plays an important role in the pathogenesis of human alcohol withdrawal [22]. Impaired cortical inhibition has been reported in persons exposed to substances of abuse, suggesting that abnormal cortical excitability may reflect changes in the brain systems of GABA and glutamate [5].

These findings are important as they indicate altered cortical excitability in drug-dependent populations and suggest that TMS can be utilized as an investigative tool to better understand the pathophysiology of addiction. Nevertheless, these studies have several limitations. First, these were exploratory studies with small samples. Second, it is difficult to determine whether alterations in the corticospinal measures are a direct result of chronic drug administration or, rather, occur due to preexisting vulnerabilities or perhaps even a combination of both. Finally, poly-substance use and/or a comorbid psychiatric condition could also exacerbate these alterations in cortical excitability.

## 4. rTMS as a Therapeutic Tool

Repetitive TMS can modulate the excitability of stimulated cortex and interconnected brain regions, even after the period of stimulation [11, 12, 29]. It has been reported that high frequency rTMS (>5 Hz) transiently increases cortical excitability [30, 31]. Changes in neurotransmission induced by rTMS have been observed in both animal [32, 33] and human studies [34, 35]. rTMS over the PFC seems to have modulator effect on mesolimbic and mesostriatal dopaminergic systems. Strafella et al. [34] found that high frequency rTMS on the prefrontal cortex in humans induces subcortical release of dopamine in caudate nucleus. Cho and Strafella [35] showed that rTMS over the left DLPFC modulates the release of dopamine in anterior cingulated cortex and orbitofrontal cortex in the same hemisphere.

The effect of rTMS on dopaminergic neurotransmission and cortical excitability suggests that this technique can be used in the study and treatment of various neuropsychiatric disorders associated with abnormal dopamine activity and altered cortical excitability, such as depression [36–39], obsessive-compulsive disorder [40, 41], schizophrenia [42–45], and drug addiction [46–56]. The efficacy of administering TMS protocols, to frontal brain areas, as a potential nonpharmacological candidate for increasing dopamine levels in the mesocorticolimbic circuitry and altering neuroadaptations induced by chronic drug use has been assessed with animal studies [5].

## 5. rTMS in the Treatment of Addiction: The Rationale and Experimental Evidence

Recent studies have begun to assess the effects of rTMS on addictive behaviours in humans. Repeated exposure to drugs can cause long-term neural adaptations in some systems. These neuroadaptations are partly associated with altered dopamine activity in the mesocorticolimbic circuitry [57, 58] and lead to an alteration of glutamate neurotransmission [59] and cortical excitability [60, 61], which have been implicated in the persistence of drug-seeking behaviours, increased difficulties regulating drug-seeking behavior, and a heightened likelihood of relapse [62].

Because rTMS can affect cortical excitability and increase the release of dopamine in the mesolimbic dopaminergic system, it is thought that repeated applications of rTMS may affect neuroadaptation induced by the chronic use of substances. In addition, rTMS can modulate neuronal activity and, thus, at least induce acute effects on circuitries that mediate different behaviours. Repeated sessions of rTMS of the PFC are, therefore, suggested to reduce drug craving, drug-seeking, and eventually drug consumption and relapse [48].

There is accumulating evidence that stimulating the dorsolateral part of the prefrontal cortex (DLPFC) may be of use in addiction treatment. DLPFC is involved in the decision-making processes [63] and these processes can be altered by rTMS [64]. Addiction is associated with increased impulsivity and willingness to take risks, which in turn can lead to impaired decision-making [65]. rTMS on the DLPFC could modulate these decision-making processes in addiction and thereby reduce impulsivity and enhance inhibitory control, which may lead to a reduction in the use of substances. Therefore, the neuropsychological assumption underlying research using rTMS in the treatment of addiction is that exciting the DLPFC by high frequency pulses should increase its activity and increase its inhibitory control function. In particular, with drug-addicted subjects, this treatment should increase DLPFC function to cope with drug craving. In fact, to date, several human studies have evaluated the effects of rTMS protocols on drug craving, a major component determining relapse, and consumption in nicotine [46–50], alcohol- [51–54], and cocaine-dependent groups [55, 56].

The following section describes studies which have explored the therapeutic potential of rTMS in reducing addictive behaviours within substance-dependent populations.

### 5.1. rTMS and Nicotine

Five papers which examined rTMS and nicotine dependence are summarized below and in Table 1.

Johann et al. [46] and Eichhammer et al. [47] investigated with exploratory studies whether high frequency rTMS of DLPFC could decrease nicotine-seeking related behaviours. In the first pilot double-blind crossover study, Johann et al. [46] investigated whether rTMS of the DLPFC could modulate levels of tobacco craving. Eleven treatment-seeking smokers under 12-hour abstinent conditions were administered either one active or one sham session of 20 Hz rTMS over the left DLPFC at 90% of MT. The session consisted of 20 trains of stimuli of 2.5 s. The levels of tobacco craving were assessed using a 100-point visual analogue scale (VAS) both 30 minutes prior to and following the rTMS treatment. rTMS significantly reduced the level of tobacco craving reported 30 minutes following the treatment [46]. These findings, therefore, motivated further investigation on the efficacy of rTMS as a potential treatment in nicotine dependence to reduce not only the level of craving but also of smoking consumption.

Following this pilot study, the same research group [47] investigated the effects of two sessions of active and sham rTMS at the same parameters with a double-blind crossover design study. Participants consisted of 14 treatment-seeking tobacco dependents. All participants were required to abstain from smoking 12 hours before the rTMS sessions. In a randomized order, each participant received 2 active trials and 2 sham stimulation sessions over 4 consecutive days. Smoking craving was measured at baseline and 30 min after the rTMS session using a VAS. In addition, the number of cigarettes freely smoked in a 6-hour time period following treatment was recorded. During this 6-hour time period, the number of cigarettes smoked following high frequency rTMS applied to the left DLPFC was significantly decreased, but craving levels remained unchanged. The authors have suggested that the evaluation of craving may have not been sensitive enough and that the sample size was probably too small to detect alterations in craving [47]. Treatment with high-frequency rTMS was, therefore, found to decrease craving level for tobacco in the pilot study, although this finding was not replicated in the second study. The second study demonstrated reduced smoking consumption following rTMS session, thus contributing to the preliminary evidence of the utility of rTMS treatment in nicotine dependence [67]. Based on these findings, the authors proposed that high frequency rTMS could have potential therapeutic value in the treatment of nicotine dependence through a reduction in the levels of craving [46] and its consumption [47].

Amiaz et al. [48] were also interested in evaluating the effects of high frequency rTMS of the left DLPFC, combined with either smoking or neutral cues, on cigarette consumption, dependence, and craving. The researchers expanded on the previous two studies by assessing whether exposure to smoking cues (prior to the stimulation) could modulate the effect of rTMS. They postulated that in addition to the effect of rTMS on dopamine transmission and cortical excitability, rTMS over the PFC may disrupt craving-related circuitries and that this effect would be prominent when rTMS is applied immediately after activation of such circuitry by smoking cues. Forty-eight heavy smokers motivated to quit smoking were recruited for the study. Participants were randomly divided into real and sham stimulation groups. Each group was subdivided randomly into two subgroups presented with either smoking-related or neutral cues just before the daily TMS intervention. Thus, there were four experimental groups: active TMS with smoking pictures, active TMS with neutral pictures, sham TMS with smoking pictures, and sham TMS with neutral pictures. The authors assessed the effects of 10 days of treatment with either active or sham 10 Hz rTMS treatment applied to the left DLPFC. Daily rTMS sessions were applied every weekday and then a maintenance phase was conducted in which rTMS sessions were less frequent. Ten daily sessions of high frequency (10 Hz) rTMS over the left DLPFC were administered in an attempt to induce long-lasting effects. Stimulation included 20 trains/day at 100% of MT and each train consisted of 50 pulses at 10 Hz with an inter-train interval of 15 s. Prior to the rTMS, participants were exposed to either smoking or neutral visual cues. Cigarette consumption was evaluated objectively by measuring cotinine (a metabolite of nicotine) urine levels before the first and the 10th treatment, and participants were administered standard questionnaires on nicotine consumption, craving, and dependence. VAS was used to evaluate levels of nicotine craving before and after the presentation of the smoking or the neutral cues and after rTMS administration. rTMS, independent of exposure to smoking pictures, reduced subjective and objective measures of cigarette consumption and nicotine dependence. It also reduced cue-induced craving and blocked the development of general craving induced by repeated presentation of smoking-related pictures. However, these effects tended to dissipate after the 10 daily sessions and the reduction in cigarette consumption was not significant 6 months after treatment termination. Interestingly, there was a trend for lower cigarette consumption in the active rTMS-smoking picture group at 6 month followup. Overall, results from this study suggested that high frequency rTMS over the DLPFC reduced cigarette consumption and nicotine dependence [48].

Wing et al. [49] examined the efficacy of high frequency rTMS for smoking cessation in treatment-seeking individuals with schizophrenia, a population of smokers who are typically highly nicotine dependent. These authors completed a 10-week, randomized, double-blind, and sham-controlled trial of rTMS (20 sessions; 5 treatments/week in weeks 1–4) as an adjunct to weekly group therapy and transdermal nicotine (TN; 21 mg) provided in weeks 3–9 in 15 heavily-dependent smokers aged 18 to 60 with schizophrenia or schizoaffective disorder. They were motivated to quit within the next month and the target quit date was the start of week 3. Subjects were randomly assigned to receive active (*N* = 6) or sham (*N* = 9) rTMS. Bilateral rTMS (randomized and counterbalanced) was administered to the DLPFC. 20 Hz stimulation was administered at 90% of the resting motor threshold for 25 trains (30 pulses/train; 30 s intertrain interval; 750 pulses/hemisphere). Sham stimulation was administered in the single-wing tilt position. Smoking (self-report and breath carbon monoxide [CO] levels), psychiatric measures (Positive and Negative Syndrome Scale [PANSS]), and adverse events were assessed weekly. Craving, using the Tiffany Questionnaire for Smoking Urges (TQSU), and withdrawal, using the Minnesota Nicotine Withdrawal Scale, were assessed pre- and post-rTMS once during each treatment week.

Consistent with findings in nonpsychiatric smokers [46], pre- and post-rTMS data collected in week 1 showed that treatment with rTMS significantly reduced craving. While there was a robust increase in craving following the rTMS session in the sham group (due to abstinence from smoking), posttreatment cravings in the active group were the same or lower than the pretreatment assessment. rTMS did not alter craving in weeks 2–4. As short-term smoking abstinence did not increase craving in these weeks, data obtained in week 1 likely provides the most sensitive measurement of rTMS effects on craving. Despite attenuation of tobacco craving, rTMS did not increase abstinence rates. An increased number of rTMS sessions may be needed for the effects on craving to translate to effects on smoking [49].

Rose et al. [50], instead, investigated the rTMS effects over superior frontal gyrus (SFG), a specific prefrontal cortex region, which supports cue-induced craving. They implemented a within-subject design to examine subjective responses to smoking versus neutral cues and to controlled presentations of cigarette smoke. Fifteen smokers were recruited for study participation and were exposed to three conditions on different days: (1) high frequency rTMS (10 Hz) over superior frontal gyrus (SFG), a specific prefrontal cortex region, which supports cue-induced craving; (2) low frequency (1 Hz) rTMS over the SFG; (3) low frequency (1 Hz) rTMS over the motor cortex (control condition). In each condition, subjects were stimulated for three periods of 2 minutes and 30 seconds at 90% MT. The researchers evaluated craving for cigarettes and they found that compared to 1 Hz rTMS to the SFG or motor cortex, 10 Hz to the SFG resulted in increased cue-induced craving but lower craving during presentation of neutral cues. Craving after smoking cue presentations was elevated in the 10 Hz SFG condition, whereas craving after neutral cue presentations was reduced. Ratings of immediate craving reduction as well as the intensity of interoceptive airway sensations were also attenuated upon smoking in the 10 Hz SFG condition. These findings support the idea that SFG plays a role in modulating craving reactivity, and that the SFG plays a role in both excitatory and inhibitory influences on craving but do not provide evidence for the utility of rTMS of the SFG for the treatment of tobacco addiction [50].

In summary, as shown in Table 1, the first four studies described above showed that high frequency rTMS of the DLPFC can attenuate nicotine consumption [47, 48] and craving [46, 49]. Rose et al. [50], instead, highlighted the excitatory and inhibitory influence of SFG on tobacco cravings but did not provide evidence for the utility of rTMS of the SFG for the treatment of tobacco addiction. However, the significance and duration of these effects are limited and further investigation is required to identify the appropriate stimulation parameters and targets. While other types of brain stimulation techniques (transcranial direct current stimulation, cranial electrostimulation, and deep brain stimulation) have been evaluated in the treatment of nicotine addiction, there is more evidence to support rTMS's potential to treat nicotine dependence [66]. According to the criteria suggested by Brainin et al. [6], the extant research on the therapeutic use of rTMS for nicotine dependence has one study in class II [48], three studies in class III [46, 47, 50], and one study in class IV [49] that showed reduction in craving, consumption, and dependence. Thus, according to the available evidence, rTMS falls within the level B recommendation as probably effective in the treatment of nicotine addiction.

### 5.2. rTMS and Alcohol

Studies of rTMS in alcohol-dependent individuals are summarized below and in Table 2.

Mishra et al. [51] published the first study about rTMS treatment of alcohol dependence. These authors studied the anticraving efficacy of high frequency rTMS of the right DLPFC in a single-blind, sham-controlled study that involved 45 patients with alcohol dependence. Participants were assigned to one of the two groups in which they received either real (*N* = 30) or sham (*N* = 15) rTMS. They were administered 10 daily sessions of high frequency (10 Hz) rTMS at 110% of MT over the right DLPFC. Each session consisted of 20 trains with 4.9 s per train and 30 s inter-train interval. The Alcohol Craving Questionnaire (ACQ-NOW) was administered in order to evaluate the extent of alcohol craving at baseline, immediately after the last rTMS and a month after the last session. There was a significant reduction in the post-rTMS ACQ-NOW total score and factor scores in the group allocated to active rTMS compared to the group allocated to sham stimulation. It was found that 10 daily sessions of high-frequency rTMS over right DLPFC had significant anticraving effects in alcohol dependence. This study supports the therapeutic potential of rTMS which, especially when combined with anticraving drugs, could represent an effective strategy in reducing craving and alcohol relapse among alcohol-dependent subjects [51].

Höppner et al. (2011) investigated the effect of high frequency rTMS of the left DLPFC compared to sham stimulation on craving and mood in alcohol-dependent women. Moreover, this study examined the impact on an attentional blink (AB) paradigm to pictures with neutral, emotional, and alcohol-related contents. Nineteen female detoxified participants were randomized either to a high frequency rTMS (20 Hz) over the left DLPFC (*N* = 10) or sham stimulations (*N* = 9) for 10 days. Alcohol craving was assessed with the Obsessive Compulsive Drinking Scale and depressive symptoms were assessed by the Hamilton Depression Rating Scale and the Beck Depression Inventory. An age-matched control group was investigated for the AB paradigm. There were no significant differences in clinical parameters such as alcohol craving or mood after real rTMS compared to sham stimulation. In the AB paradigm, real stimulated participants detected alcohol-related T2 targets incorrectly in comparison to the sham stimulated and control subjects. Although there were no differences in craving and mood after real high frequency rTMS compared to sham stimulation, an interesting difference between the real and the sham stimulated group and controls was found in the AB paradigm, suggesting an increase of the AB effect to alcohol-related pictures after real stimulation [53].

Herremans et al. (2012) performed a prospective, single-blind, and sham-controlled study in order to investigate the effect of single high frequency rTMS session of the right DLPFC on alcohol craving in the community. Participants (*N* = 36) were hospitalized alcohol-dependent patients. After successful detoxification, participants were allocated to receive one active or one sham rTMS session. The rTMS session was administered on friday before the weekend at home. The rTMS session consisted of 40 trains of 1.9 s at 20 Hz at 110% of MT with a 12 s inter-train interval. The obsessive-compulsive drinking scale (OCDS) was administered to assess the intensity of alcohol craving just before and after the rTMS session (on friday), on saturday and sunday during the weekend at home, and on monday (when the participants returned to the hospital). One high frequency rTMS session delivered to the right DLPFC did not lead to changes in craving (neither immediately after the stimulation session nor in participants' natural environment during the weekend). This study found that application of a single rTMS session had no significant effect on alcohol craving [52].

In a case report [54], a 48-year-old woman with a 23-year heavy drinking history was stimulated with low-frequency rTMS targeting the dorsal anterior cingulated cortex (dACC) using a double cone coil in an attempt to suppress very severe alcohol craving; 1 Hz rTMS (50% of the machine's intensity, total of 600 pulses) was delivered for 3 weeks to the medial frontal cortex. It was a combined rTMS neuroimaging study: functional magnetic resonance imaging (fMRI) and resting state electroencephalography (EEG) were performed before rTMS treatments, after successful rTMS and after unsuccessful rTMS with relapse. Blood alcohol volumes were acquired on random days during the treatment course. Alcohol cravings were measured daily by a VAS during a cue exposure. Repetitive rTMS reduced alcohol consumption for the duration of the treatment. Symptoms of withdrawal and cravings were also reduced up to 3 months. This reduction was also reflected with the change in fMRI and EEG activity. After 3 months, the patient relapsed and was treated with 1 week of rTMS. These effects lasted for 3 weeks until the patient relapsed again and the patient became unresponsive to the rTMS treatment [54].

In conclusion, 10 daily sessions of high-frequency rTMS over right DLPFC had significant anticraving effects in alcohol dependence [51], while a single rTMS session had no significant effect on immediate and long-term craving; there were no reduction in craving after high frequency rTMS over left DLPFC [53]; repetitive rTMS targeting the dACC using a double cone coil reduced immediate alcohol craving and consumption [54]. These studies together suggest that rTMS may play a therapeutic role in alcohol dependence, though the evidence is still preliminary [68], and also indicate that one single session could be too short to alter alcohol craving in alcohol-dependent patients. As we can see in Table 2, stimulation parameters, such as frequency, % of MT, train duration, intertrain interval, and area of stimulation, differ significantly among studies. Based on these findings, Herremans and Baeken (2012) suggested the evaluation of multiple rTMS sessions in larger, randomized, and sham-controlled population samples. Furthermore, randomized controlled studies should be done to evaluate whether patients need stimulation with high or low frequency [69].

According to the criteria suggested by Brainin et al. [6], rTMS therapy for alcohol dependence includes one case report [54] (class IV) and three controlled clinical trials (class III) [51–53], two of which reported no effect on craving and one showed reduction in craving. Thus, there is inadequate evidence to confer a level of recommendation for its effectiveness.

### 5.3. rTMS and Cocaine

Camprodon and colleagues [55] conducted a preliminary study to examine rTMS as a potential treatment for the craving experienced by cocaine-dependent individuals. They investigated whether a single session of rTMS over DLPFC could reduce cocaine craving among six male participants with cocaine dependence. Secondary endpoints were changes in anxiety, happiness, sadness, and discomfort. In this randomized crossover design, participants were administered two sessions of high frequency (10 Hz) rTMS at 90% of MT, to the right and left DLPFC, with a week break between the two sessions. Patients were asked to complete a set of 15 visual analogue scales (VAS) ranging from “not at all” to “more than ever.” Each VAS evaluated one of the primary or secondary endpoints on three occasions: 10 minutes before the intervention and immediately after and 4 hours after rTMS session.

The authors found that a single session of high frequency rTMS to the right DLPFC, but not to the left DLPFC, decreased level of craving for cocaine. This is a transient effect that resolves within 4 h after stimulation.

The resulted anxiety significantly reduced after right-sided stimulation. Happiness was increased after right-sided and sadness after left-sided stimulation. Discomfort was increased equally by left- and right-sided rTMS and the interaction term was not significant, providing a useful control for the nonspecific effects of the stimulation.

This research provided the first demonstration that high frequency rTMS applied over the right DLPFC is effective in reducing craving associated with chronic use of cocaine [55]. Also Politi et al. (2008) studied the effects of rTMS over the DLPFC on cocaine cravings among 36 cocaine-dependent participants studied after detoxification. Ten daily sessions of high frequency (15 Hz) rTMS over the left DLPFC at 100% of MT were administered. The participants underwent daily clinical assessment of symptoms associated with cocaine craving. These authors found that the daily sessions of high frequency rTMS of the left DLPFC reduced cocaine craving. Cocaine cravings reduced gradually throughout the sessions [56].

As shown in Table 3, both these studies [55, 56], using different paradigms and assessment tools, suggest that rTMS over the DLPFC can potentially provide an effective therapeutic intervention for cocaine craving and addiction. There was a discrepancy in the laterality of the findings. Further research is required on the optimal stimulation patterns and on the exact brain region to stimulate. Future studies that assess cocaine intake after treatment are also required. According to the criteria suggested by Brainin et al. [6], studies of rTMS therapy for cocaine dependence includes one clinical trial (class III) [55] and one study rated in class IV [56] that showed reductions in craving. Thus, there is inadequate evidence to confer a level of recommendation for the effectiveness of this treatment.

## 6. Conclusion and Future Studies

In summary, in the research that evaluated the therapeutic use of rTMS in addiction we have one clinical trial classified in class II [48] that showed reduction in craving, consumption, and dependence, five trials classified in class III that support the effectiveness of rTMS [46, 47, 50, 51, 55], and two studies in the same class that reported the ineffectiveness of this treatment [52, 53]. Thus, based on the available evidence, rTMS can be classified as probably effective in the treatment of addiction. At the moment, the best level of evidence of the effectiveness of rTMS is in the treatment of nicotine dependence.

The effects of rTMS sessions on drug craving and consumption provide evidence and support for further TMS studies in the field of addiction research. Most of the studies that assessed the therapeutic potential of rTMS to treat alcohol, nicotine, and cocaine addiction were performed using a figure-of-eight coil targeting DLPFC. These studies show that high frequency rTMS over the DLPFC can reduce craving and consumption in nicotine, alcohol, and cocaine-dependent populations. These studies are still few and have also methodological limitations: they were exploratory in nature and consist of relatively small sample sizes. Future research should identify the optimal parameters (appropriate target, intensity, frequency, and length) of stimulation in rTMS studies for the most effective and safe treatment of drug addiction. Future studies with multiple rTMS sessions in larger, randomized, and sham-controlled population samples are required.

It is important to note that none of these studies demonstrated complete abstinence from substance use and few studies [47, 53] evaluated craving in the natural environment of the patients. It also could be that subjective craving assessment is not that reliable [53]. In addition, the period of rTMS to maintain the gains produced need to be examined with longer follow-up studies.

Fitzgerald and Daskalakis review reported that high-frequency rTMS applied to the left DLPFC is an effective treatment for patients with major depressive disorder [70], although the review included only two studies [53, 55] that took in consideration that targeting the DLPFC might affect mood. Camprodon et al. [55] found that in cocaine addicted individuals happiness was increased after right-sided and sadness after left-sided stimulation [55]. These findings were in contrast with those from studies of patients with depression, who tend to show the opposite lateralization [70, 71]. Instead, Höppner et al. (2011) did not find significant differences in mood after real rTMS over the left DLPFC in individuals with alcohol dependence [53]. Thus, more research is needed on the impact of rTMS on mood in individuals with alcohol and drug dependence. More studies that measure psychological and neurophysiological variables, such as frontal activation tasks, quantitative EEG, evoked potentials, and functional magnetic resonance, along with rTMS in addiction are required for more comprehensive assessment of the treatment effects. Given the critical interaction between brain stimulation and cognitive activation revealed in some studies (e.g., Amiaz et al., 2009), it is possible that a combination of brain stimulation and cognitive behavioural therapies would produce a promising clinical outcome [5]. These findings might translate across the spectrum of addictive disorders [72].

## Figures and Tables

**Table 1:** Summary of the studies on rTMS in the treatment of nicotine addiction.

Study	*N* subjects (active, sham)	Place of stimulation	Number of sessions	Length	Frequency	Intensity of stimulation	Sham stimulation	Assessment	Findings
Johann et al. (2003) [46]	11	Left DLPFC	1 session	20 trains of 2.5 s	20 Hz	90% MT	Yes	Craving, assessed by VAS	Reduction in craving

Eichhammer et al. (2003) [47]	14	Left DLPFC	4 sessions (2 active and 2 sham)	20 trains of 2.5 s	20 Hz	90% MT	Yes (between the group)	Craving, assessed by VAS; cigarettes number	Reduction in consumption

Amiaz et al. (2009) [48]	22, 26	Left DLPFC	10 sessions	20 trains of 5 s	10 Hz	100% MT	Yes	Craving, assessed VAS; cigarettes number	Reduction in craving, consumption, and dependence

Wing et al. (2012) [49, 66]	6, 9	Bilateral DLPFC	20 sessions	50 trains	20 Hz	90% MT	Yes	Craving, assessed by TQSU	Reduction in craving

Rose et al. (2011) [50]	15	SFG	3 sessions (1 active 1 Hz, 1 active 10 Hz, 1 MOC)	2.5 min each session (total period of stimulation: 7.5 min)	1 Hz or 10 Hz	90% MT	Yes	Craving, assessed by Shiffman-Jarvik questionnaire and cigarette evaluation questionnaire	Reduction in craving (10 Hz)

DLPFC: dorsolateral prefrontal cortex; SFG: superior frontal gyrus; MOC: motor cortex; MT: motor threshold; VAS: visual analogue scale; TQSU: Tiffany Questionnaire for Smoking Urges.

**Table 2:** Summary of the studies on rTMS in the treatment of alcohol addiction.

Study	*N* subjects (active, sham)	Alcohol use status	Place of stimulation	Number of sessions	Length	Frequency	Intensity of stimulation	Sham stimulation	Assessment	Findings
Mishra et al. (2010) [51]	30, 15	After detoxification	Right DLPFC	10 daily sessions	20 trains of 4.9 s	10 Hz	110% MT	Yes	Craving, assessed by ACQ-NOW	Reduction in immediate craving; no effect on craving after 4 weeks

Höppner et al. (2011) [53]	10, 9 (females)	14 days after detoxification	Left DLPFC	10 daily sessions	1000 pulses	20 Hz	90% MT	Yes	Craving, assessed by OCDS; depressive symptoms, assessed by BDI; AB for neutral and alcohol related pictures	No reduction in craving and no effect on mood; increase AB for alcohol related pictures

Herremans et al. (2012) [52]	36	After detoxification	Right DLPFC	1 session	40 trains of 1.9 s	20 Hz	110% MT	Yes	Craving, assessed by OCDS	No effect on immediate and long-term craving

de Ridder et al. (2011) [54]	1 (female)	Active drinking period	dACC	daily sessions during 5 weeks	600 pulses	1 Hz	50% machine output	No	Craving, assessed by VAS	Reduction in immediate craving and consumption; relapse after 3 months with increased craving after 3 months

DLPFC: dorsolateral prefrontal cortex; dACC: Dorsal anterior cingulated cortex; MT: motor threshold; VAS: visual analogue scale; ACQ-NOW: Alcohol Craving Questionnaire; OCDS: obsessive compulsive drinking scale; BDI: Beck Depression Inventory; AB: attentional blink.

**Table 3:** Summary of the studies on rTMS in the treatment of cocaine addiction.

Study	*N* subjects	Place of stimulation	Number of sessions	Length	Frequency	Intensity of stimulation	Sham stimulation	Assessment	Findings
Camprodon et al. (2007) [55]	6	Bilateral DLPFC	2 sessions	20 trains of 10 s	10 Hz	90% MT	No	Craving, anxiety, happiness, sadness, and discomfort, assessed by VAS	Reduction in craving (with right DLPFC rTMS); reduction in anxiety after right-sided rTMS; increase in happiness after right-sided and in sadness after left-sided rTMS; increase in discomfort equally by left- and right-sided stimulation

Politi et al. (2008) [56]	36	Left DLPFC	10 daily sessions	20 trains of 2 s	15 Hz	100% MT	No	Clinical assessment of craving related symptoms	Reduction in craving

DLPFC: dorsolateral prefrontal cortex; MT: motor threshold; VAS: visual analogue scale.
